# Correspondence

**Published:** 1883-07

**Authors:** Ehrick Parmly

**Affiliations:** New York


### CORRESPONDENCE.

NEW YORK, April 21, 1883.

DEAR DOCTOR:—Since my communication on Abortion in Cows was read before the Farmers' Club, the following interesting cases have occurred. Two cows sent to the farm for service were returned to their owners after passing one heat, as probably being in calf. Their exposure or stay in my herd was respectively 23 and 26 days, and both prior to the second abortion, which occurred December 5th. One of these cows has been since October 26th in a stable 30 miles distant, with a farrow cow. Both were kept tied in their stalls. No jumping and consequent strain to excite suspicion in that direction. (I mention this, as some men are averse to admitting any cause but mechanical or external injury.) She aborted at 6 months on March 27th. The other case aborted at 6 months 9 days, 40 miles distant from the farm in an opposite direction.

Before the above mishaps occurrred, I warned the owners of all cows that had been on the farm to separate them from other pregnant cows, and to keep apart for several weeks after aborting, or even should they carry to term. I have other cases to hear from. No mishap has occurred as yet to any animal that came to the farm for service and went away at once.

Yours truly,

EHRICK PARMLY, M.D.

